# Clonal rearrangements and Malignant Clones in Peripheral T-cell Lymphoma

**Published:** 2015

**Authors:** Yu. V. Sidorova, N. G. Chernova, N. V. Ryzhikova, S. Yu. Smirnova, M. N. Sinicina, Yu. E. Vinogradova, H. L. Julhakyan, A. M. Kovrigina, E. E. Zvonkov, A. B. Sudarikov

**Affiliations:** National Hematology Research Center of the Ministry of Health, Novy Zykovski lane 4a, 125167, Moscow, Russia; I.M. Sechenov Moscow State Medical University, Department of Hospital Therapy №2, B. Pirogovskaya, 4, 119435 Moscow, Russia

**Keywords:** peripheral T-cell lymphoma, PCR, gene rearrangement of T cell receptor, T-lymphocytes clonality

## Abstract

Aim: To assess the feasibility and informative value of T-cell clonality testing in peripheral T-cell lymphoma (PTCL). Patients and methods: Biopsies of involved sites, blood, and bone marrow samples from 30 PTCL patients are included in the study. Rearranged *TCRG *and *TCRB *gene fragments were PCR-amplified according to the BIOMED-2 protocol and analyzed by capillary electrophoresis on ABI PRISM 3130 (Applied Biosystems). Results: *TCRG *and *TCRB *gene clonality assay was valuable in confirming diagnosis in 97% of PTCL patients. T-cell clonality assay performed on blood or bone marrow samples reaffirmed lymphoma in 93% of cases, whereas morphological methods were informative in 73% of cases only. We observed multiple *TCRG* and *TCRB *gene rearrangements, loss of certain clones in the course of the disease, as well as acquisition of new clones in 63% of PTCL cases, which can be attributed to the genetic instability of the tumor. Conclusion: *TCRG* and *TCRB *gene clonality assay is beneficial for the diagnosis of PTCL. However, the presence of multiple clonal rearrangements should be considered. Clonal evolution in PTCL, particularly acquisition of new clones, should not be treated as a second tumor. Multiple *TCRG *and *TCRB *gene rearrangements may interfere with minimal residual disease monitoring in PTCL.

## INTRODUCTION

**Fig. 1:**
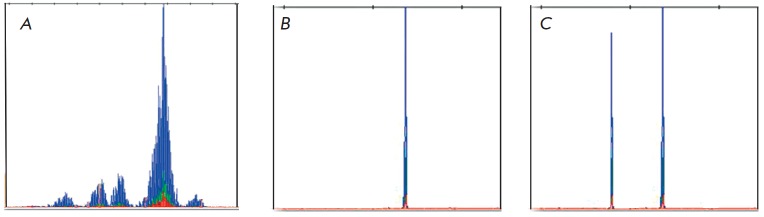
Example of TCRG clonality testing: a) polyclonal, b) monoclonal (monoallelic rearrangement), c) monoclonal (biallelic rearrangement)

Peripheral T-cell lymphoma, not otherwise specified (PTCL-NOS) is a heterogeneous group of lymphomas with a mature immunophenotype of peripheral (postthymic) T-lymphocytes. This diagnosis covers more than 29% of T-cell lymphomas that do not belong to other nosological forms and is a diagnosis by exclusion [1, 2]. Clinically, the disease is aggressive (five-year overall survival rate is less than 32%), often advanced (69% of patients are diagnosed at stages III/IV) with extranodal sites being involved (bone marrow, skin, subcutaneous tissue, and lungs) [3]. It is believed that the morphological substrates of the tumors are T-lymphocytes of mature T-cells immunophenotype with a αβ-variant of the T-cell surface receptor (TCRαβ) and CD2+, CD3+, CD5+, CD7+, CD4+ or CD8+ markers, whose expression displays signs of aberrance (loss of one or more of them). Most often, PTCL-NOS has a CD4+/CD8-immunophenotype, less commonly a CD4-/CD8+ one. In some peripheral T-cell lymphomas, the expression of T-cell markers on the surface is limited, e.g. only CD2 or CD3. In addition, a small number of PTCL-NOS are γδ-T-lymphocytes lymphomas that cannot be classified as hepatolienal γδ-lymphomas or γδ-variant of large granular lymphocytes leukemia based on clinical and morphological data [3-6]. A study of clonal rearrangements of T-cell receptor genes in PTCL-NOS confirms T-cell clonality in complex diagnostic cases and proves the presence of a tumor [7-11]. The method consists of PCR amplification and analysis of the genes of the V-D-J-segments junction region of the T-cell receptors δ (*TCRD*), γ (*TCRG*), and β (*TCRB*). This region has a unique nucleotide sequence in each normal T-lymphocyte. A fragment analysis of amplification products derived from healthy tissue reveals a lot of peaks with a Gaussian distribution of their lengths (*Fig.1A*). Monoclonal samples with the same length of PCR products are present as a single peak (monoallelic rearrangement, *Fig. 1B*) or as two peaks (biallelic rearrangement, *Fig. 1B*).

**Fig. 2:**
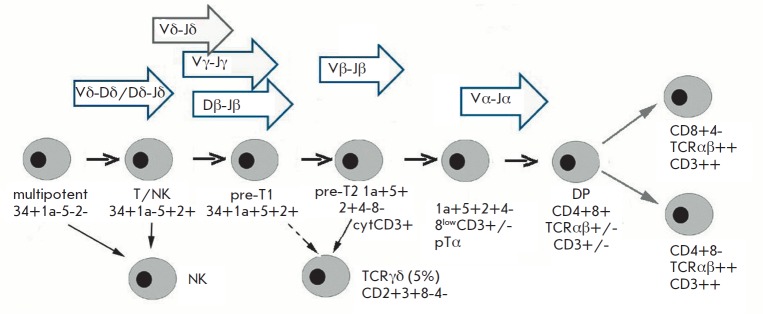
Early stages of T-cell development. Sequential rearrangements in δ, γ, β, and α chains of TCR genes. DP – double positive cells

The length of the monoclonal PCR product is unique for the tumor clone and is identical in all affected tissues of a patient. Detection of a clone, for example, in bone marrow aspirate, indicates bone marrow involvement. Furthermore, the nature of rearrangements can reveal the degree of a lymphoid tumor maturity. Rearrangements during normal maturation of T-lymphocytes occur in sequence: *TCRD *(Vδ–Dδ, Dδ–Dδ, Dδ–Jδ, Vδ–Jδ) gene locus is rearranged first, followed by *TCRG *(Vγ–Jγ) and incomplete rearrangement of *TCRB *(Dβ–Jβ). Complete rearrangements of *TCRB *(Vβ–Jβ) and *TCR*α-locus (Vα–Jα) (*Fig. 2*) occur later [12, 13]. Since *TCR*δ (TCRD) genes are located inside *TCR*α-locus, they are cut out during *TCR*α rearrangement. Therefore, the entire range of clonal rearrangements observed in lymphomas can be divided into early and more mature ones. If a tumor contains *TCRD *and *TCRG *loci clonal products, incomplete rearrangement of Dβ–Jβ, and no complete Vβ‑Jβ rearrangements of *TCRB *genes, this indicates an early nature of the rearrangements, which corresponds to a tumor of γδ-T-lymphocytes. More commonly, a tumor has a more mature spectrum of rearrangements: Vγ–Jγ, Dβ–Jβ, and Vβ–Jβ rearrangements present simultaneously, which is typical of most TCRαβ-lymphomas, including PTCL-NOS. According to published data, clonal rearrangements of the *TCRG *and *TCRB *genes are present in 81–94% and 96% of PTCL-NOS, respectively [7, 8].

## MATERIALS AND METHODS

A retrospective analysis of clonality studies results in PTCL-NOS over the last 10 years (2005‑2015) has been conducted in the National Hematology Research Center of the Russian Ministry of Health (hereinafter the NHRC).

**Patients and samples**

**Table 1:** Data of laboratory diagnostics

No	Major immunophenotypical characteristics of the tumor	Lesion volume	Stage	BM PCR	Total number of clonal rearrangements^***^			Qty clones^**^	Qty tissues studied
					TCRG, TCRD*	TCRB Dβ–Jβ	TCRB Vβ–Jβ		
1	LN CD3m+CD4+CD8-CD30-GranzBCD5- CD57-CXCL13-CD7+	LN	I	nm	3 Vγ–Jγ	3 Dβ–Jβ	0	2	1
2	Spl CD3m+CD4+CD8+CD5+TIA- 1+GranzB+CD7+CD56+	BM Liv Spl GI	IV	+	2 Vγ–Jγ	3 Dβ–Jβ	1Vβ–Jβ	3	5
3	Spl CD3m+CD4- CD8+CD2+CD7+CD30-CD10-CD5+	BM Spl	IV	+	1 Vγ-Jγ 3TCRD	2 Dβ–Jβ	5 Vβ–Jβ	4	2
4	BM CD3m+CD4+CD8+CD10-CD1a- CD5+CD7+	BM Spl	IV	+	6 Vγ–Jγ	4 Dβ–Jβ	5 Vβ–Jβ	4	2
5	BM CD3e-CD4-CD8+(part)CD7-CD2- CD1a-CD5-CD56-CD30- Spl CD3e+CD4-CD8-CD7-CD2- CD1a-CD5-CD30-ALK-GranzB	BM Spl	IV	+	3 Vγ–Jγ	3 Dβ–Jβ	3Vβ–Jβ	3	3
6	LN CD3m+CD4+CD8- CD45RO+CD2+CD7-CD30-ALK-	BM LN	IV	+	3 Vγ–Jγ	nd	nd	2	1
7	LN CD3m+CD4-CD8+CDRO+CD30- CD15-CD23-CD56-	BM LN Spl Sto	IV	+	3 Vγ–Jγ	1 Dβ–Jβ	2Vβ–Jβ	2	5
8	LN CD3m+CD4+CD8-CD 5+CD10- CD23-	BM LN Skin Ton	III	+	5Vγ–Jγ	1 Dβ–Jβ	3Vβ–Jβ	4	9
9	LN CD3m+CD4- CD8+CD2+CD5+CD30+	BM LN Skin Lar Ton	IV	+	8Vγ–Jγ	3 Dβ–Jβ	3Vβ–Jβ	7	6
10	LN CD3m+CD4-CD8+CD5+TIA-1+	BM LN GI MG Lung	IV	+	2Vγ–Jγ	3 Dβ–Jβ	3Vβ–Jβ	3	6
11	LN CD3m+CD4+CD8- CD2+CD5+CD7+CD15-CD1a-	BM LN Lung Sto Skin Liv Spl NL	III	+	4Vγ–Jγ	2 Dβ-Jβ	2 Vβ–Jβ	5	7
12	LN CD3m+CD4+CD8- CD30+GranzB-EMA+	BM LN MG Lung Ton NL	IV	+	3 Vγ–Jγ	2 Dβ-Jβ	1 Vβ–Jβ	2	2
13	Spl CD3m+CD4-CD8-TIA-I+	BM Spl Liv	IV	+	8Vγ–Jγ	2 Dβ–Jβ	4Vβ–Jβ	4	4
14	LN CD3m+CD4+CD8+CD7+CD2+CD30- NK-	BM LN Skin	IV	+	2 Vγ–Jγ	3 Dβ–Jβ	1 Vβ–Jβ	2	2
15	Med CD3m+CD4+CD8-CD30-ALK-	Mediastinum	IE	nm	2 Vγ–Jγ	1Dβ–Jβ	1 Vβ–Jβ	1	1
16	Lung CD3m+CD4+CD8- CD45RO+CD5+CD7+CD30-CD10- CD23-	LN Lung	IE	+	3Vγ–Jγ	1Dβ–Jβ	2Vβ–Jβ	2	3
17	LN CD3m+CD4+CD8- CD30+CD33+CD56-	Med LN	III	+	3Vγ–Jγ	2Dβ–Jβ	1Vβ–Jβ	2	2
18	LN CD3m+CD4-CD8+CD5+CD7+	LN	III	+	2Vγ–Jγ	0	2Vβ–Jβ	1	2
19	LN CD3m+CD4+CD8- CD5+CD4+CD10-ALK-	LN Spl	III	+?	2Vγ–Jγ	0	1Vβ–Jβ	1	2
20	Spl CD3c+CD4-CD8-CD1a- CD2+CD5-CD7-CD4-CD8- CD16+CD56+	BM Spl	IV	+	2Vγ–Jγ	1Dβ–Jβ	2Vβ–Jβ	2	2
21	LN CD3m+CD4-CD8-CD30-CD15- CD5+CD7+NK-CD2+GranzB-	BM LN	IV	+	2Vγ–Jγ	1Dβ–Jβ	2Vβ–Jβ	2	2
22	LN CD3m+CD4-CD8+CD30-CD10- CD15-CD23-	BM LN	IV	+	1Vγ–Jγ	0	2Vβ–Jβ	1	1
23	Orbit CD3m+CD4+CD8+CD5+CD7+TIA- 1+CD10-CD30-CD56-LPM-1-CD23- ALK	Soft tissues of the orbirt	IE	Nm	1Vγ–Jγ	1Dβ–Jβ	1Vβ–Jβ	1	1
24	Spl CD3m+CD4+CD8- CD2+CD5+CD7+CD56+TIA-1+	BM Spl	IV	+	2Vγ–Jγ	1Dβ–Jβ	2Vβ–Jβ	1	2
25	LN CD3m+CD4+CD8- CD2+CD7+GranzB+CD30-CXCL13- PD1–LMP1-	Spl LN	III	-	0	0	0	0	2
26	LN CD3m+CD4+CD8- CD2+CD5+CD7+CD30-ALK– GranzB-EMA-CD56-CD57-	BM LN	IV	-	0	2 Dβ–Jβ (doubt)	1Vβ–Jβ (doubt)	1	2
27	LN CD3e-CD4-CD8-CD10-CD5- CD23-CD30-ALK-LCA+CD2+CD7+ GranzB-	BM LN Skin	IV	+	0	1Dβ–Jβ (BM)	0	1	3
28	BM CD2+CD3+CD5-CD7+-CD4-CD8- CD16+56+cytCD3TCRγδ+ Spl CD3e+ (m+cyt) CD4-CD8-CD5-CD7- TIA-I+CD56+	BM Spl	IV	+	2Vγ–Jγ 3TCRD	2Dβ–Jβ	0	1	2
29	Blood CD3+TCRαβ+CD4+CD8+CD5+CD7+	BM Spl	IV	+	1Vγ–Jγ	0	2Vβ–Jβ	1	1
30	BM 30% cellsCD3+CD4-CD8-CD5- CD2+	BM Spl	IV	+	3 Vγ–Jγ	2Dβ–Jβ	1Vβ–Jβ	2	2

Note. LN, lymph node; Spl, spleen; BM, bone marrow; GI, intestine; Sto, stomach, Lar, larynx, Liv, liver; Ton, tonsils; Lung, lung; MG, mammary gland; NL, neuroleukemia; Med, mediastinum.

TCRD^*^, the study of TCRD genes was conducted in two patients with suspicion of γδ-T-cell lymphoma.

Number of clones^**^, the minimum number of tumor clones in a patient, based on the number of clonal rearrangements in a single locus and the appearance (changing) of clonal products in various tissues. Cases with appearance (change) of clonal products are underlined. For explanation, see Results and Discussion sections.

The total number of clonal rearrangements^***^, total number of clonal rearrangements (peaks) observed in all tissues examined; nm, no material, nd, no data; doubt, an uncertain picture.

The set of patients consisted of 30 people (15 men and 15 women, median age 56 years (32–75)). The disease stage was determined according to the Ann-Arbor classification (1971); bone marrow involvement was considered to be stage IV. Four patients were diagnosed with stage I; four, with stage III; and 22, with stage IV. Lymph nodes were involved in 18 (60%) patients; bone marrow, in 22 (73%); spleen, in 14 (47%); skin, in 5 (17%); gastrointestinal tract, in 4 (13%); lungs, in 4 (13%); tonsils, in 3 (10%); liver, in 3 (10%); mediastinum, in 2 (7%); meninges (neuroleukemia), in 2 (7%); mammary gland, in 2 (7% ); and soft tissues of the orbit, in 1 (3%) (*Table 1*). Histological and immunohistochemical studies were performed in the NHRC Department of Pathology; and molecular and genetic studies of clonality, in the NHRC Laboratory of Molecular Hematology.

**Isolation of DNA from tissues**

Leukocytes and DNA from blood and bone marrow samples were isolated as described [14]. For isolation of DNA from tissue embedded into a paraffin block, five 5-μm sections were collected in Eppendorf tubes. The tissue was dewaxed by heating [15, 16]. Freshly frozen tissue for DNA extraction was thawed, and a 1×1×1 mm piece was cut out. DNA was isolated by the method based on tissue dissolution in concentrated ammonia with subsequent neutralization with glacial acetic acid and salting-out of proteins [17]. DNA concentration was determined using a UV spectrophotometer. DNA samples were stored at –20°C.

**Studies of TCR gene rearrangements by PCR and fragment analysis**

T-cell clonality was assessed using multiplex BIOMED-2 primers systems for fragment analysis [13] based on the rearrangements of the *TCRG *(Vγ–Jγ) and *TCRB *(Vβ–Jβ, Dβ–Jβ) genes. In the case of γδ-Tcell lymphomas, *TCRD *genes rearrangements were also analyzed. Multiplex amplification of *TCRD *genes was performed in duplicate tubes according to the BIOMED-2 protocol, Tube A and Tube B, and *TCRB* gene amplification, in three tubes, Tube A, Tube B, and Tube C (see description of the reactions in *Table 2*). Primers produced by Syntol (Russia) were used for amplification of the *TCRD*, *TCRG *genes. The reaction mixture in a final volume of 20 μl included 100 ng of DNA, 10 μl of a 2 × PCR mixture (PCR Master Mix Promega), and 5 pmol of each primer. *TCRB *genes were amplified using a commercial TCRB Gene Clonality Assay ABI Fluorescence Detection kit (Invivoscribe Technologies) and AmpliTaq Gold DNA polymerase (Applied Biosystems) according to the manufacturers’ instructions. PCR conditions were as follows: pre-denaturation at 95°C (5 min); 35 cycles at 92°C (35 seconds), 60°C (35 seconds), 72°C (35); and final elongation at 72°C (10 min). PCR was performed using an automated DNA Engine thermocycler (BioRad, Hercules, USA).

**Table 2:** Sets of PCR primers used for the BIOMED-2 protocol for the TCRD, TCRG, TCRB genes

Set of primers (the name of the tubes)	Forward primers	Reverse primers	Length of the product, bp
TCRD Tube A	Dδ2,Vδ1,Vδ2,Vδ3,Vδ4, Vδ5,Vδ6	Jδ1FAM Jδ2R6G Jδ3TAMRA Jδ4ROX	130–280
TCRD Tube B	Dδ2,Vδ1,Vδ2,Vδ3,Vδ4, Vδ5, Vδ6	Dδ3FAM	190–280
TCRG	Vγ1f, Vγ9, Vγ10, Vγ11	Jγ1/2FAM Jp1/2FAM	100–250
TCRB Tube A	Vβ2–Vβ24 (23 primers)	Jβ1.1–Jβ1.6HEX (6 primers) Jβ2.2, Jβ2.6, Jβ2.7 FAM (3 primers)	240–280
TCRB TubeB	Vβ2–Vβ24 (23 primers)	Jβ2.1, Jβ2.3, Jβ2.4, Jβ2.5 FAM (4 primers)	240–280
TCRB TubeC	Dβ1,Dβ2	Jβ1.1–Jβ1.6HEX Jβ2.1–Jβ2.7FAM (13 primers)	170–210 (Dβ2) 290–310 (Dβ1)

An automatic ABI PRISM 3130 Genetic Analyzer (Applied Biosystems, USA) was used for the fragment analysis of PCR products. To perform it, 2 μL of a 20-fold diluted PCR product was mixed with 10 μL of formamide (Applied Biosystems) and 0.04 μL of GeneScan 500-LIS Size Standard (Applied Biosystems). After denaturation at 95°C for 3 minutes and subsequent cooling, 10 μL of the mixture was added to a well of a 96-well plate and high resolution capillary electrophoresis was performed on a POP-4 polymer (Applied Biosystems). The fluorescence of the amplificates and their profile (length distribution) were analyzed by the GeneMapper software v. 4.0 (Applied Biosystems).

**Statistical analysis**

To compare the results obtained by the two methods, the Spearman rank correlation coefficient was calculated using the following formula: *r*_s_ = 1 – 6Σ*d*^2^/(*N*^3^ –* N*), where *N *is the sample size; d is the difference between the ranks for each member of the sample; and r_s_ is the Spearman coefficient.

## RESULTS

*TCR *gene clonality was detected in 29 of the 30 patients (97%): for *TCRG *genes, in 27 of the 30 (90%); and for* TCRB *genes, in 29 of the 30 (97%). In some PTCL samples, the rearrangement was detected in only one of the loci, *TCRG *or *TCRB*, which is due to abnormal differentiation of tumor cells and is often associated with an immature and aberrant immunophenotype. For example, in patient 27, whose bone marrow had only one clonal Dβ–Jβ rearrangement, the surface of the tumor cells did not have CD3, CD5, CD4, or CD8, but only CD2 and CD7. The most likely explanation for the lack of clonal peaks in the study of *TCR *genes (patient 25) is the small number of tumor cells in the sample (many reactive T-lymphocytes). PCR detected bone marrow involvement or the presence of clonal lymphocytes in the blood in 93% (25 out of 27) of patients (*Table 1*). The clonal rearrangements in bone marrow were not detected only in patients 25 and 26, for whom clonal peaks were also absent or doubtful in the lymph nodes. Morphological methods failed to detect bone marrow involvement in four patients (№ 16–19), whereas PCR detected clonal cells in their bone marrow. Detection of clonal rearrangements of *TCRG *genes in the bone marrow or blood is considered to be a poor prognostic factor in PTCL [18]. In most patients, we observed multiple (more than two) clonal rearrangements in one locus. Tumor lymphocytes clone may have a rearrangement on one chromosome (monoallelic rearrangement) or on two homologous chromosomes (biallelic rearrangement). Therefore, only one or two clonal peaks can be detected for each gene (*TCRD* or *TCRG *or *TCRB*) in one tumor clone.

**Fig. 3:**
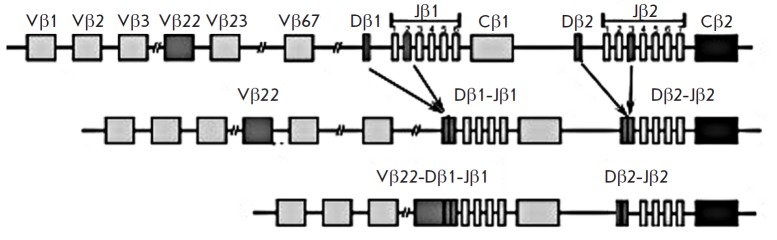
Additional Dβ2–Jβ rearrangement acquisition diagram. Figure shows TCRB locus withVβ22–Dβ1‑Jβ1 and Dβ2– Jβ2 fusions

Additional Dβ2–Jβ rearrangement (*Fig. 3*) is described as an exception [19, 20]. In this case, an additional clonal peak in a range from 170–210 bp was observed in Tube C during fragment analysis after amplification of *TCRB *genes (*Table 2*).

In 13 out of the 30 (43%) patients, three or more clonal peaks were detected in one *TCR *gene locus in at least one tissue (10 patients, in the *TCRG *locus; 11, in the *TCRB *locus). Multiple (three or more) clonal peaks were detected at the same frequency in bone marrow, lymph nodes, and/or spleen (in 10 out of 13 patients). In theory, the “extra” peaks can be attributed to reactive T-cells, but we faced a completely different situation. In 63% of the patients (15 out of 24) in whom we had analyzed several tissues, we observed the appearance of new clonal peaks and new clones in various tissues. The clonal rearrangements reported earlier were either not detected or partially preserved. There was a correlation between observations for the *TCRG *and* TCRB *genes: i.e., the appearance of a new clonal peak of *TCRG *genes was usually accompanied by the identification of a new clonal rearrangement of *TCRB *genes. This pattern can only be explained by the presence of several tumor clones and their different representations in the tissues and organs. In total, several clones were detected in 19 of the 30 patients (63%). The number of clones ranged from two to seven (*Table 1*). There was no correlation with age (*p *= 0.43) or the stage of the disease (*p *= 0.29). Identification of several clones correlated with the number of analyzed tissues (r_s_ = 0.6,* p* < 0.0005). This phenomenon might have been overlooked in other patients due to the low number of investigated tissues. The most representative examples of clonality studies in PTCL are given below.

**Fig. 4:**
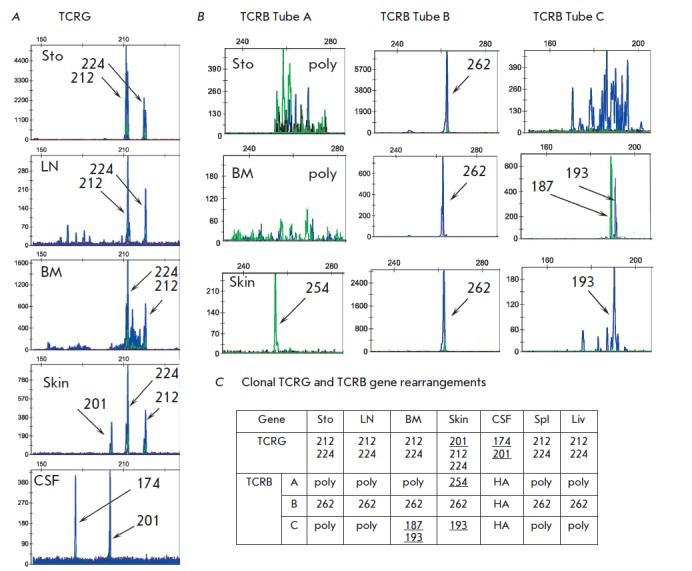
Clonal TCR rearrangements found in case 11.A, TCRG pattern; B, TCRB pattern; C, Summary Table. Sto, stomach, BM, bone marrow, LN, lymph node, CSF, poly, polyclonal case, NA, no amplification. New clonal products are underlined

**Patient 11** (*Fig. 4*). At the time of the diagnosis, Patient 11 displayed a pattern typical of mature T-cell lymphoma: biallelic rearrangement of *TCRG *genes (212 and 224 bp) and complete rearrangement of *TCRB* genes (Tube B, 262 bp). Bone marrow examination revealed two more clones with incomplete rearrangement of *TCRB *genes (Tube C 187 and 193 bp). Progression of the disease leads to skin lesions, which were accompanied by one or two new clonal rearrangements of *TCRG *genes (201 bp) and complete rearrangement of *TCRB *genes (Tube A 254 bp). Further progress leads to the development of neuroleukemia. The cerebrospinal fluid (CSF) lacked the clones previously detected in the tumor (*TCRG *212 and 224 bp) but contained a clone with rearrangement of *TCRG *genes, 201 bp in length, and a new clone with rearrangement of *TCRG* genes, 174 bp in length. After 11 months, the patient underwent splenectomy and liver biopsy due to growing cytopenia. The clonal rearrangement pattern in spleen and liver is identical to the original one in the stomach and lymph node. Therefore, Patient 11 has a total of four rearrangements of the *TCRG *gene: two full rearrangements of *TCRB *genes and two incomplete rearrangements of the *TCRB *gene. The dynamics of appearance of new rearrangements during the progression of the disease indicates five or more different tumor clones.

**Fig. 5:**
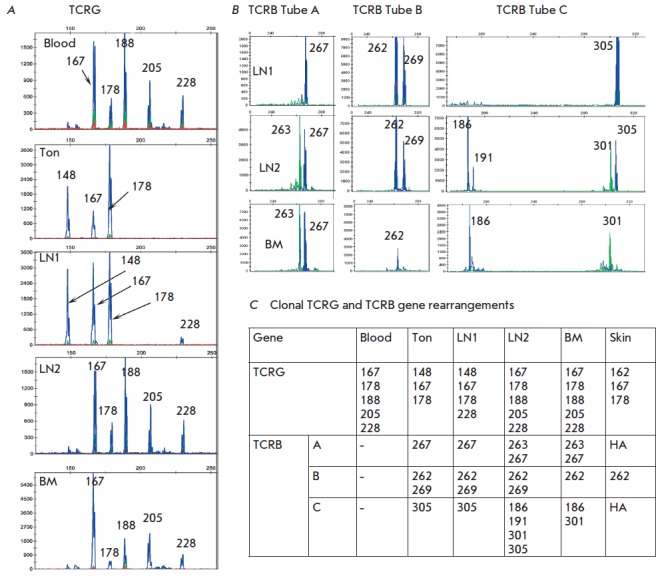
Clonal TCR rearrangements found in case 9.A, TCRG pattern; B, TCRB pattern; C, Summary table. Ton, tonsil; BM, bone marrow; LN, lymph node; NA, no amplification

**Patient 9** (*Fig. 5*). Patient 9 had multiple clonal rearrangements of the *TCRG *and *TCRB *genes with a varying presence of clones in different tissues and organs. The primary diagnosis included examination of the blood, bone marrow, tonsil biopsy and lymph node 1. Five or more rearrangements of the *TCRG *and* TCRB *genes were detected in the blood and bone marrow. Tonsil and lymph node 1 contained three and four clonal rearrangements of *TCRG*, respectively, and four rearrangements of the *TCRB *gene. Lymph node 2 and skin biopsies were performed with progression of the disease. The lymph node biopsy revealed five rearrangements of the *TCRG *gene and eight of the *TCRB* genes. Except for the rearrangements of *TCRB *(A), 267 bp, and *TCRB *(B), 262 bp, that are present in most tissues simultaneously, other rearrangements of *TCRB* genes were randomly distributed and belonged to different tumor clones. The data of molecular clonality studies indicate the presence of seven or more tumor clones in this patient.

**Fig. 6:**
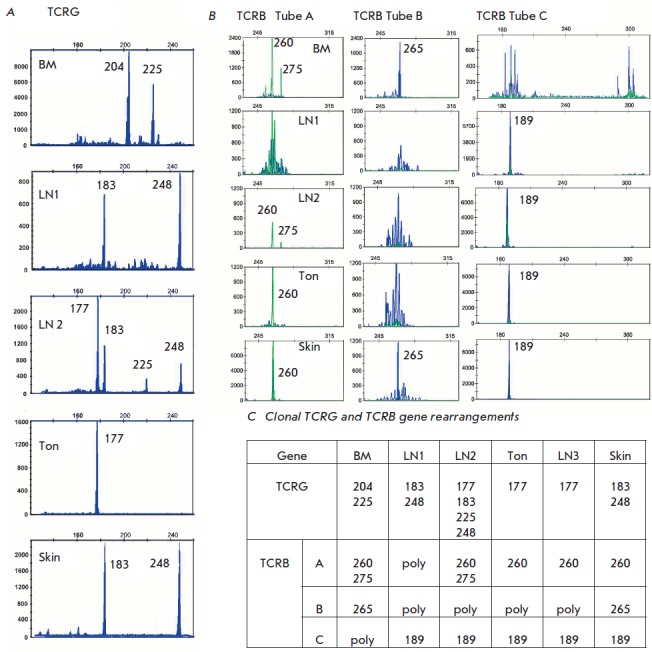
Clonal TCR rearrangements found in case 8.A, TCRG pattern; B, TCRB pattern; C, Summary table. Ton, tonsil; BM, bone marrow; LN, lymph node; poly, polyclonal case

**Patient 8** (*Fig. 6*). Patient 8 had five different rearrangements of *TCRG *genes (177, 183, 204, 225, 248 bp) with different clones present in different studied tissues. We found four rearrangements of *TCRB*: three complete rearrangements (Tubes A and B) and one incomplete Dβ2–Jβ2 (Tube C). In this case, there was no clear connection between the *TCRG *and *TCRB *genes rearrangements. For example, the lymph node 1 and skin predominantly had *TCRG *gene rearrangements, 183 and 248 bp in size, however, the pattern for *TCRB* was different. The number of clones in this patient was four or higher, but it is possible that the number of clones could be much higher. For example, the clones with only a *TCRG *or *TCRB *rearrangement, or clones with rearrangements of *TCRG *177 bp and *TCRB *260 bp, *TCRG *177 bp and *TCRB *189 bp, etc.

## DISCUSSION

The presence of several tumor clones described in acute lymphoblastic leukemia (ALL) is attributed to an “ongoing” process of rearrangement of immunoglobulins and *TCR *genes in early progenitor cells [21-24]. Clonal products sequencing during ALL manifestation and in relapse showed that clones with incomplete rearrangements of the *TCRD *and *TCRB *genes and their derivatives with complete clonal rearrangements are simultaneously present in ALL. Furthermore, in some cases complete rearrangement is modified. The *V *gene is replaced with another (upstream) gene or the rearrangement is completely replaced by another one of the upstream *V *and downstream *J *genes, i.e. by the genes that are distal to the previous rearrangement. In some cases, there is a deletion and disappearance of the *TCR* gene rearrangement in relapse.

We believe that the molecular events that occur in ALL can also occur in PTCL. In tumor cells, the control mechanisms which are responsible for preventing further restructuring of a locus after a productive rearrangement are disrupted, which leads to increased activity of the *RAG1 *and *RAG2 *enzymes, changes in chromatin organization, etc. It is possible that in PTCL rearrangements can be replaced with new ones and/or that an incomplete Dβ–Jβ one can be replaced with a complete Vβ–Jβ one. In addition, there is general chromosomal instability, which can lead to deletions or duplications of the *TCR *genes locus. The deletion of the locus may trigger further rearrangements on homologous chromosomes. Complex chromosomal changes, including triploidy, tetraploidy, loss of chromosomes, 7q trisomy, and translocations involving *TCR *genes loci (14q11, 7q34-35, 7p13-21) are described in various peripheral T-cell lymphomas [25-27]. In any case, clonal rearrangements are common markers that indicate the heterogeneity of the tumor and clonal evolution during the progression of the disease. We have observed “multiclonality” in most patients, but it is unclear whether this phenomenon is present only in PTCL. It is possible that some other lymphomas also have high clonal heterogeneity, but their “visualization” requires different approaches and methods.

## CONCLUSIONS

Studies of *TCRG *and *TCRB *genes clonality effectively prove the presence of a tumor in the majority (97%) of PTCL patients. PCR revealed bone marrow involvement and/or presence of clonal lymphocytes in the blood in the majority (93%) of patients, whereas in morphological studies bone marrow involvement was confirmed only in 73% of the patients. A specific pattern of rearrangements in PTCL (multiple rearrangements of the *TCRG *and *TCRB *genes, loss and gain of new ones, presence of several clones of a tumor) was observed in the majority of patients (63%) and should certainly be taken into account when using this method for diagnostic purposes. The emergence of new clonal peaks (clones) should not be considered to be the emergence of a new tumor. In addition, multiple rearrangements may interfere with minimal residual disease monitoring in PTCL.

## Abbreviations

PTCL-NOS: peripheral T-cell lymphoma, not otherwise specified
TCR: T-cell receptor
TCRG: T-cell receptor gamma
TCRB: T-cell receptor beta
TCRD: T-cell receptor delta
CD: cluster of differentiation
